# Cigarette smoking is associated with higher thyroid hormone and lower TSH levels: the PREVEND study

**DOI:** 10.1007/s12020-019-02125-2

**Published:** 2019-11-09

**Authors:** Eke G. Gruppen, Jenny Kootstra-Ros, Anneke Muller Kobold, Margery A. Connelly, Daan Touw, Jens H. J. Bos, Eelko Hak, Thera P. Links, Stephan J. L. Bakker, Robin P. F. Dullaart

**Affiliations:** 1 Department of Nephrology, University of Groningen, University Medical Center Groningen, Groningen, The Netherlands; 2 Department of Endocrinology, University of Groningen, University Medical Center Groningen, Groningen, The Netherlands; 3 Department of Laboratory Medicine, University of Groningen, University Medical Center Groningen, Groningen, The Netherlands; 4 Laboratory Corporation of America Holdings (LabCorp), Morrisville, NC USA; 5 Department of Clinical Pharmacy and Pharmacology, University of Groningen, University Medical Center Groningen, Groningen, The Netherlands; 6 Clinical Pharmacoepidemiology, University of Groningen, Groningen Research Institute of Pharmacy, Unit PharmacoEpidemiology and PharmacoEconomics, Groningen, The Netherlands

**Keywords:** Alcohol consumption, Antiperoxidase autoantibodies, Cigarette smoking, FT4, FT3, TSH

## Abstract

**Purpose:**

The extent to which smoking is associated with thyroid stimulating hormone (TSH), free thyroxine (FT4), and free triiodothyronine (FT3) when taking account of clinical variables including alcohol consumption is unclear. We aimed to determine associations of TSH, FT4, and FT3 levels with current smoking.

**Methods:**

A cross-sectional study was performed in 5766 euthyroid participants (Prevention of Renal and Vascular End-Stage Disease cohort). Current smoking was determined by self-report, categorized as never, former, and current (≤20 and >20 cigarettes per day). Smoke exposure was determined by urinary cotinine.

**Results:**

Current smoking of ≤20 and >20 cigarettes per day was associated with lower TSH and higher FT3 levels. FT4 levels were higher in subjects smoking <20 cigarettes per day vs. never and former smokers. Current smokers also consumed more alcohol. Multivariable linear regression analyses adjusted for age, sex, anti-TPO autoantibody positivity, alcohol consumption, and other variables demonstrated that lower TSH, higher FT4 and higher FT3 were associated with smoking ≤20 cigarettes per day vs. subjects who never smoked (*P* < 0.001, *P* = 0.018, and *P* < 0.001, respectively) without a further significant incremental effect of smoking >20 cigarettes per day. In agreement, TSH was inversely, whereas FT4 and FT3 levels were positively associated with urinary cotinine (*P* < 0.001 for each). In contrast, alcohol consumption >30 g per day conferred higher TSH and lower FT3 levels.

**Conclusions:**

Cigarette smoking is associated with modestly higher FT4 and FT3, and lower TSH levels, partly opposing effects of alcohol consumption.

## Introduction

Disorders in thyroid function are very common in the general population [1, 2], and it is increasingly appreciated that variations in thyroid function, as inferred from circulating levels of thyroid stimulating hormone (TSH), free thyroxine (FT4), and free triiodothyronine (FT3) within their respective reference ranges, may impact on a number of health issues including atherosclerosis susceptibility [3–6]. Although not unequivocally reported, it is also noteworthy that variations in thyroid function within the reference range could affect life expectancy [7, 8]. It is, therefore, relevant to better understand the impact of life style factors on thyroid function status.

It has long been recognized that cigarette smoking may affect thyroid function, conceivably attributable to exposure to toxic metabolites, enhanced sympathetic nervous activity, or by affecting thyroid-directed autoimmune responses [4, 9, 10]. In the eighties of the last century, it was already documented that smoking cessation elicits a rise in TSH and a decrease in T4 [11]. Indeed, with some exceptions, evidence has accumulated since that cigarette smoking is associated lower TSH and higher FT4 levels, whereas T3 and FT3 were also found to be higher or unaltered [9, 10, 12, 13]. In addition, using serum or urinary cotinine as a measure of nicotine exposure it was found that smoke exposure is associated with lower TSH levels [14, 15].

Among other life style factors, alcohol consumption has been suggested to lower FT4 [16]. Moreover, FT4 and FT3 levels are decreased in alcohol abusers after alcohol withdrawal [17, 18]. Given that cigarette smoking may frequently coincide with more heavy alcohol consumption [18, 19], it is plausible to take account of alcohol use when evaluating the association of cigarette smoking with thyroid hormone levels. However, epidemiological surveys on the effect of smoking on thyroid function parameters thus far did not comprehensively assess the extent to which such associations are independent of alcohol consumption and other relevant characteristics.

For this reason the present study was initiated to determine the association of cigarette smoking assessed by self-report as well as by urinary cotinine concentrations with TSH, FT4, and FT3 taking account of alcohol consumption, as well as other clinical and laboratory covariates in the Prevention of Renal and Vascular End-Stage Disease (PREVEND) cohort, a large and comprehensively characterized population from the north of the Netherlands.

## Materials and methods

### Study population

**Fig. 1:**
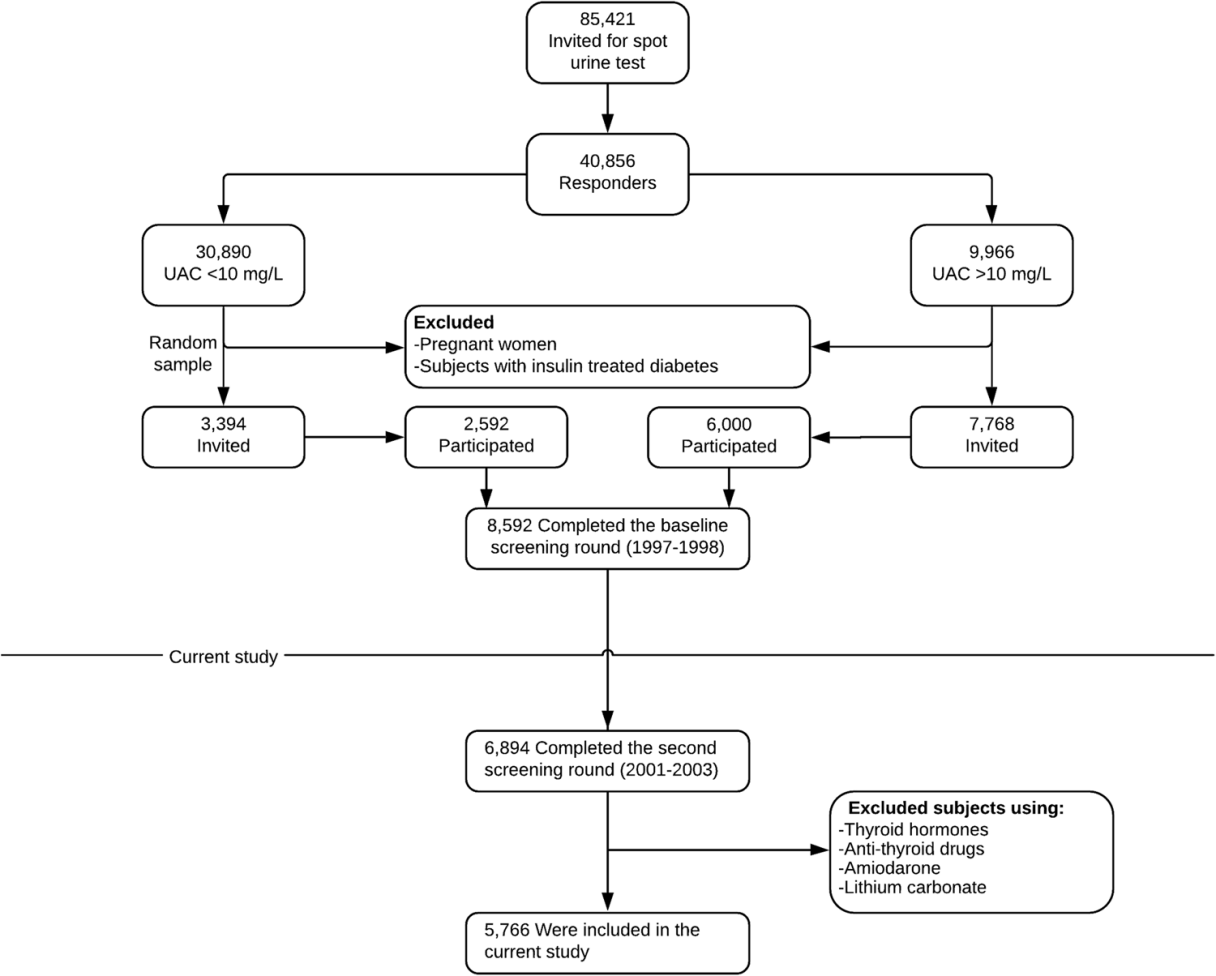
Flowchart depicting PREVEND participants included or excluded for the purposes of this study

The study was performed in the frame of the PREVEND cohort study, which investigates vascular and renal damage among predominantly white inhabitants of the city of Groningen, The Netherlands, as reported in detail elsewhere [19–23]. The study was approved by the Medical Ethics Committee of the University Medical Center Groningen and conformed to the principles outlined in the Declaration of Helsinki. All participants gave written informed consent. Briefly, in 1997–1998, all inhabitants of the city of Groningen, aged 28–75 years, were sent a short questionnaire on demographics and cardiovascular morbidity and a vial to collect an early morning urine sample (*n* = 85,421). Pregnant women and diabetic subjects using insulin were excluded. Altogether 40,856 subjects responded (47.8%), and their urinary albumin concentration was determined. Subjects with a urinary albumin concentration ≥10 mg/L (*n* = 7768) were invited to participate, of whom 6000 were enrolled. In addition, a randomly selected group with a urinary albumin concentration <10 mg/L (*n* = 3394) was invited to participate in the cohort, of whom 2592 were enrolled. The initial study population was comprised of 8592 subjects who completed the total screening program. For the present study, we used data from participants who completed the second screening round (2001–2003; *n* = 6894), excluding those with missing values for smoking behavior, thyroid function tests, as well as those who were not euthyroid (see Measurements and Definitions) and those using thyroid hormones, antithyroid drugs, amiodarone, or lithium carbonate. This left a cohort of 5766 participants with sufficient information for analysis (Fig. 1).

### Measurements and definitions

Euthyroidism was defined as TSH, FT4, and FT3 levels all within the euthyroid reference range (TSH: 0.27–4.20 mU/L; FT4: 12–22 pmol/L; FT3:3.1–6.8 pmol/L). Cigarette smoking was estimated by questionnaire, and was categorized as (i) never; (ii) former; (iii) current ≤20 cigarettes per day, and (IV) current >20 cigarettes per day. In addition, urinary cotinine concentrations were measured. Former smokers were subjects who were nonsmokers at the time of the second screening round but had ever smoked in their life; current smokers were those who reported smoking at this screening. Alcohol intake was estimated by using predefined categories covering a time period of one month [19, 22]. Alcohol intake was then calculated as alcoholic drinks per day with one drink being considered to be equivalent to 10 g of alcohol, regardless of the type of beverage [19]. Alcohol intake was categorized in (i) no/rarely; (ii) 0.1–10 g per day (occasional to light); (iii) 10–30 g per day (moderate), and (IV) >30 g per day (heavy).

The presence of a self-reported history of myocardial infarction, percutaneous coronary intervention, coronary artery bypass surgery, stroke, or the diagnosis of narrowing of one or both carotid arteries was defined as cardiovascular disease. Type 2 diabetes mellitus was defined as a fasting serum glucose concentration >7.0 mmol/L, a nonfasting serum glucose concentration >11.1 mmol/L, a self-report of a physician diagnosis, or the use of glucose-lowering drugs [23].

Information on medication use was combined with information from IADB.nl, a data base containing information of prescribed medication in public pharmacies in The Netherlands (http://www.iadb.nl/) [24]. Body mass index (BMI) was calculated as weight (kg) divided by height squared (m). Blood pressure was measured using an automatic device (Dinamap XL model 9300 series; Johnson-Johnson Medical, Tampa, FL, USA). Urinary albumin excretion (UAE) was measured in two consecutive 24 h urine collections after oral and written instruction. The participants were asked to avoid heavy exercise as much as possible during the urine collection. Subjects were also instructed to postpone their urine collection in case of urinary tract infection, menstruation, or fever. The UAE results were averaged for analysis. Estimated glomerular filtration rate (eGFR) was calculated applying the combined creatinine cystatin C-based Chronic Kidney Disease Epidemiology Collaboration equation [25]. The participants were instructed to have their blood samples being taken in the morning after an overnight fasting period.

### Laboratory methods

Venous blood samples were obtained after 15 min rest. Venous plasma and serum samples were obtained by centrifugation at 1400 × *g* for 15 min at 4 °C. Serum glucose was measured shortly after blood sampling. Ethylenediaminetetraacetic acid anticoagulated plasma and heparinized plasma and serum was obtained by centrifugation at 4 °C, and stored at −80 °C until analysis. Cotinine concentrations were measured with with Enzyme Multiplied Immunoassay Technique on the Abbott Architect c8000 system (Abbott Laboratories, Abbott Park, IL, USA). Serum TSH, FT4, FT3, and antithyroid peroxidase (anti-TPO) autoantibodies were determined using the Roche Modular E170 Analyzer electrochemiluminescent immunoassays (Roche Diagnostics, Mannheim, Germany). Anti-TPO autoantibodies were considered positive using a cut-off value as indicated by the supplier (≥35 kU/L). Twenty-four hour urinary cotinine levels were measured with Enzyme Multiplied Immunoassay Technique on the Abbott Architect c8000 system (*Abbott* Laboratories, *Abbott* Park, IL).

Total cholesterol was measured on an automatic analyzer (MEGA; Merck, Darmstadt, Germany) using the CHOD–PAP-method. Glucose was measured using Kodak Ektachem dry chemistry (Eastman Kodak, Rochester, New York, USA). HDL cholesterol and triglycerides were determined using the nuclear magnetic resonance (NMR)-derived LP4 lipoprotein profile deconvolution algorithm as described [26]. To this end NMR spectra were acquired on a Vantera^®^ Clinical Analyzer at LabCorp (Morrisville, NC, USA). Serum creatinine was measured by an enzymatic method on a RocheModular analyzer (Roche Diagnostics, Mannheim, Germany). Serum cystatin C was measured by Gentian Cystatin C Immunoassay (Gentian AS, Moss, Norway) on a Modular analyzer (Roche Diagnostics). Urinary albumin was measured by nephelometry (Dade Behring Diagnostic, Marburg, Germany) [19].

### Statistical analyses

Data analysis was performed using IBM SPSS software (version 23.0, SPSS Inc., USA). Normally distributed data are given as mean ± SD and nonparametrically distributed data are presented as median (interquartile range). Categorical variables are given as numbers and %. Anti-TPO autoantibody titers (as continuous variable), TSH, triglycerides, urine cotinine, and UAE were natural logarithm (log_e_) transformed in order to achieve approximately normal distributions. Differences in continuous variables between categories of cigarette smoking were determined by Analysis of Variance with subsequent Bonferroni procedure to correct for multiple comparisons. Differences in TSH, FT4, and FT3 according to anti-TPO antibody status were determined by unpaired *T*-tests. Proportions of dichotomous variables across categories of cigarette smoking were determined by *χ*^2^ tests. Pearson correlation coefficients were calculated to determine univariate relationships. Multivariable linear regression analyses were used to discern the independent association of current cigarette smoking with TSH, FT4, and FT3. In these analyses, the category “never smoked” was used as the reference category. In these analyses, we also used categorized alcohol consumption as variable with the category “no/rarely” as reference. In addition, analyses were repeated with urinary cotinine data instead of self-reported smoking status. In these analyses, urinary cotinine concentrations of 0 were set at 0.01 to allow for log_e_ transformation. Standardized regression coefficients (*β*) were used to discern the strength of associations of current smoking (≤ and >20 cigarettes per day) with TSH, FT4, and FT3. Two-sided *P*-values < 0.05 were considered significant.

## Results

The clinical and laboratory characteristics of the participants are shown in Table 1. Of the 5766 participants, 29.0% had never smoked, 42.2% were former smokers, 24.9% smoked ≤20 cigarettes per day, and 3.9% smoked >20 cigarettes per day. Urinary cotinine concentrations, measured in 5722 subjects, were highest in subjects who smoked >20 cigarettes per day, and virtually absent in never smokers and former smokers. Current smokers were younger than former smokers and more likely to be men. There were also differences in hemodynamics across the four groups, with systolic blood pressure being lower in current smokers vs. former smokers and pulse rate being highest in subjects who smoked >20 cigarettes per day. The most frequent use of antihypertensive and lipid lowering drug was observed in the group of former smokers. Alcohol consumption was lowest in subjects who never smoked and highest in subjects who smoked >20 cigarettes per day. eGFR was lowest and UAE was highest in former smokers. Total cholesterol was highest and HDL cholesterol was lowest in subjects who smoked ≤20 cigarettes per day, whereas triglycerides were lowest in subjects who smoked >20 cigarettes per day. As shown in Table 1, TSH levels were lower in both groups of smokers vs. never and former smokers. FT4 levels were higher in subjects who smoked ≤20 cigarettes per day vs. never and former smokers, whereas FT3 levels were highest in both groups of smokers vs. never and former smokers. Anti-TPO antibody titers were highest in current smokers. The highest prevalence of positive anti-TPO antibodies was observed in subjects who smoked >20 cigarettes per day.

**Table 1:** Clinical and laboratory characteristics according to smoking status in 5766 participants of the PREVEND study

	Never smokers	Former smokers	Current smokers ≤20 cigarettes per day	Current smokers >20 cigarettes per day	*P*-value
Urine cotinine (ng/mL)^*^	0 [0–0]	0 [0–0]	1191 [660–1721]	1744 [1310–2296]	<0.001
Number (%)	1670 (29.0)	2436 (42.2)	1433 (24.9)	227 (3.9)	
Age (years)	51 ± 12	56 ± 12	52 ± 11^d^	50 ± 8^d^	<0.001
Sex (M: *n*; %)	750 (44.9)	1408 (57.8)	728 (50.8)	112 (49.3)	<0.001
Systolic blood pressure (mm Hg)	125 ± 8	129 ± 19	123 ± 18^d^	124 ± 16^e^	<0.001
Diastolic blood pressure (mm Hg)	72 ± 9	75 ± 9	73 ± 9^d^	75 ± 8^b^	<0.001
Pulse pressure (mm Hg)	53 ± 13	54 ± 14	51 ± 11^a,d^	53 ± 12^b,d^	<0.001
Pulse rate (beats/min)	68 ± 10	68 ± 10	69 ± 10^c,d^	74 ± 10^a,d^	<0.001
BMI (kg/m^2^)	26.5 ± 4.4	27.2 ± 4.3	27.2 ± 4.3^a^	26.5 ± 4.5	<0.001
Diabetes (*n*; %)	91 (5.4)	167 (6.9)	69 (4.8)	12 (5.3)	0.050
History of CVD (*n*; %)	46 (2.8)	211 (8.7)	106 (7.4)	8 (3.5)	<0.001
Antihypertensive drugs (*n*; %)	274 (16.4)	642 (26.4)	236 (16.5)	39 (17.2)	<0.001
Glucose lowering drugs (*n*; %)	54 (3.2)	94 (3.9)	39 (2.7)	3 (1.3)	0.082
Lipid lowering drugs (*n*; %)	102 (6.1)	267 (11.0)	140 (9.8)	22 (9.7)	<0.001
Alcohol consumption (g/day)					<0.001
0/rarely (*n*; %)	515 (30.8)	503 (20.6)	333 (23.2)	54 (23.8)	
0.1–10 (*n*; %)	352 (21.1)	377 (15.5)	219 (15.3)	14 (6.2)	
10–30 (*n*; %)	557 (33.4)	778 (31.9)	458 (32.0)	56 (24.7)	
>30 (*n*; %)	246 (14.7)	778 (31.9)	423 (29.5)	103 (45.3)	
Glucose (mmol/L)	5.0 ± 1.2	5.1 ± 1.2	4.9 ± 1.1^d^	5.0 ± 1.2	
eGFR (ml/min/1.73 m^2^)	95 ± 16	91 ± 18	91 ± 18^a^	97 ± 14^d^	<0.001
UAE (mg/24 h)	7.8 (5.8–2.8)	9.2 (6.2–18.1)	8.8 (6.015.9)^a,f^	7.8 (5.8–12.8)^a^	<0.001
Total cholesterol (mmol/L)	5.3 ± 1.0	5.4 ± 1.0	5.5 ± 1.0^a^	5.3 ± 1.0^a,f^	<0.001
HDL cholesterol (mmol/L)	1.3 ± 0.3	1.3 ± 0.3^b^	1.2 ± 0.3^a,e^	1.3 ± 0.3^b,e^	<0.001
Triglycerides (mmol/L)	1.3 (0.7–1.4)	1.1 (0.8–1.6)	1.2 (0.9–1.7)^b^	1.0 (0.7–1.4)^b^	<0.001
TSH (mU/L)	1.68 (1.17–2.39)	1.62 (1.14–2.21)	1.44 (1.00–1.96)^a,d^	1.48 (0.94–1.91)^a,d^	<0.001
FT4 (pmol/L)	15.7 ± 1.9	15.7 ± 1.9	15.9 ± 1.9^c,d^	15.9 ± 2.0	<0.001
FT3 (pmol/L)	4.9 ± 0.5	4.8 ± 0.5	4.9 ± 0.5^a,d^	5.0 ± 0.5^a,d^	<0.001
Anti-TPO autoantibody titer (IU/mL)	9.8 (7.8–13.2)	9.6 (7.6–12.9)	11.4 (9.3–15.0)^a,d^	12.7 (10.7–16.3)^a,d^	<0.001
Anti-TPO autoantibody positive (*n*; %)	150 (8.9)	163 (6.7)	110 (7.7)	22 (9.7)	0.036

Smoking status is categorized as never; former and current, which is divided in <20 and ≥20 cigarettes per day. Alcohol consumption is categorized in 0/rarely; 0.10; 10–30 and >30 g per day. Ten grams of alcohol is equivalent to one alcoholic drink. Anti-TPO autoantibodies were not available four subjects. Data are given in mean ± SD, medians (interquartile ranges), or in numbers (%). Between group differences are determined by analysis of variance. Bonferroni method is applied to correct for multiple comparisons. *P*-value: *P*-value by analysis of variance or by *χ*^2^-analysis. *anti*-*TPO* antithyroid peroxidase, *BMI* body mass index, *CVD* cardiovascular disease, *eGFR* estimated glomerular filtration rate, *FT*4 free thyroxine, *FT*3 freetriiodothyronine, *HDL* high density lipoproteins, *TSH* thyroid stimulating hormone, *UAE* urinary albumin excretion

Asterisk indicates that urine cotinine was measured in 5722 participants

^a^*P* < 0.001

^b^*P* < 0.01

^c^*P* < 0.05 vs. never smokers

^d^*P* < 0.001

^e^*P* < 0.01

^f^*P* < 0.05 vs. former smokers

TSH was higher, whereas FT4 and FT3 levels were lower in women vs. men (women (*n* = 2768): TSH 1.65 (1.14–2.32) mU/L, FT4 15.5 ± 1.8 pmol/L, and FT3 4.7 ± 0.5 pmol/L vs. men (*n* = 3008): TSH 1.50 (1.08–2.08) mU/L, FT4 16.0 ± 1.9 pmol/L, and FT3 4.7 ± 0.5 pmol/L, *P* < 0.001 for each comparison between women and men). Positive anti-TPO antibodies were more frequently present in women vs. men (Supplemental Table 1). Moreover, when thyroid function was assessed according to positive anti-TPO antibody status, TSH was found to be higher, whereas FT4 and FT3 were lower in subjects who were positive for anti-TPO antibodies (Supplemental Table 1).

**Fig. 2:**
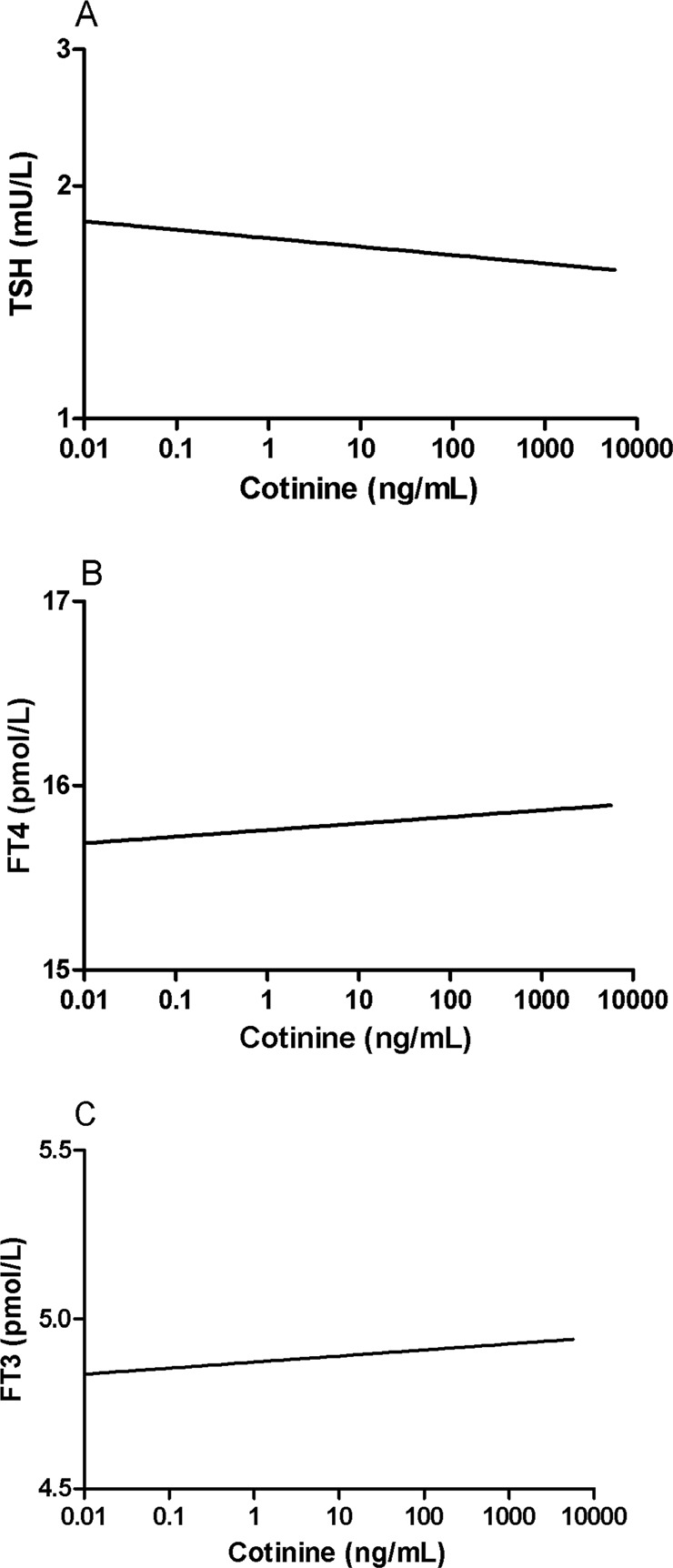
Relationship of thyroid stimulating hormone (TSH) **a**, free thyroxine (FT4) **b**, and free triiodothyronine **c** with urinary cotinine excretion in 5722 participants of the PREVEND study. TSH and urinary cotinine are log_e_ transformed. Univariate correlation coefficients: TSH with urinary cotinine: *r* = −0.118, *P* < 0.001; FT4 with urinary cotinine: *r* = 0.044, *P* = 0.001; FT3 with urinary cotinine: *r* = 0.079, *P* < 0.001

Multivariable linear regression analyses demonstrated that TSH levels were associated inversely with current cigarette smoking independent of age and sex with the never smokers as reference category (Table 2; model 1). Conversely, there were positive associations of FT4 (Table 2, model 2) and FT3 (Table 2, model 3) with current cigarette smoking. However, as indicated by the standardized regression coefficients (β), these associations were not stronger in subjects who smoked >20 cigarettes per day vs. subjects who smoked ≤20 cigarettes per day. We also performed analysis with urinary cotinine concentrations as objective reflection of smoke exposure (Table 3). The univariate inverse correlations of TSH with urinary cotinine are shown in Fig. 2a, whereas the positive correlations with FT4 and FT3 are shown in Fig. 2b, c, respectively.

**Table 2:** Multivariable linear regression analyses demonstrating associations of thyroid function variables with cigarette smoking adjusted for age and sex in 5766 participants of the PREVEND study

	Model 1: TSH		Model 2: FT4		Model 3: FT3	
	*β*	*P*-value	*β*	*P*-value	*β*	*P*-value
Age	−0.021	<0.001	−0.028	0.036	−0.167	<0.001
Sex (M vs. F)	−0.075	<0.001	0.117	<0.001	0.286	<0.001
Smoking						
Never (reference) former	−0.037	0.020	0.026	0.10	−0.008	0.59
Current ≤20 cigarettes per day	−0.140	<0.001	0.043	0.006	0.055	<0.001
Current >20 cigarettes per day	−0.083	<0.001	0.018	0.18	0.058	<0.001

Smoking status is categorized as never (reference category); former and current, which is divided in <20 and ≥20 cigarettes per day. β: standardized regression coefficients

*F* female, *FT*4 free thyroxine, *FT*3 free triiodothyronine, *M* male, *TSH* thyroid stimulating hormone. TSH is log_e_ transformed

**Table 3:** Multivariable linear regression analyses demonstrating associations of thyroid function variables with cotinine levels adjusted for age and sex in 5722 participants of the PREVEND study

	Model 1: TSH		Model 2: FT4		Model 3: FT3	
	*β*	*P*-value	*β*	*P*-value	*β*	*P*-value
Age	−0.007	0.56	−0.033	0.007	−0.137	<0.001
Sex (M vs. F)	0.068	<0.001	−0.111	<0.001	−0.207	<0.001
Urinary cotinine	−0.085	<0.001	0.038	0.002	0.045	<0.001

β: standardized regression coefficients. TSH and urinary cotinine are log_e_ transformed

*F* female, *FT*4 free thyroxine, *FT*3 free triiodothyronine, *M* male, *TSH* thyroid stimulating hormone

Given many differences in clinical and laboratory variables across the four categories of smoking behavior (Table 1), we next performed multivariable linear regression analyses in which we took account of a large number of relevant clinical and laboratory variables. As shown in Table 4, these analyses also showed that TSH levels remained inversely, whereas FT4 and FT3 levels remained associated positively with current smoking. These analyses also did not demonstrate stronger associations in subjects who smoked >20 cigarettes per day vs. subjects who smoked ≤20 cigarettes per day. Notably, these analyses also demonstrated lower TSH, higher FT4, and higher FT3 levels in men, independent of anti-TPO antibody status, as well as inverse associations of TSH, FT4, and FT3 with age. FT4 was positively and FT3 was inversely associated with BMI. Of further note, TSH levels were positively, whereas FT3 levels were inversely associated with heavy alcohol consumption. Table 5 shows the multivariable adjusted results of thyroid function with urine cotinine concentrations with an inverse association with TSH and positive associations with FT4 and FT3, comparable to the results with self-reported smoking status.

**Table 4:** Multivariable linear regression analyses demonstrating independent associations of thyroid function variables with cigarette smoking in 5766 participants of the PREVEND study

	Model 1: TSH		Model 2: FT4		Model 3: FT3	
	β	*P*-value	β	*P*-value	β	*P*-value
Age	−0.087	<0.001	−0.098	<0.001	−0.221	<0.001
Sex (M vs. F)	−0.051	0.001	0.126	<0.001	0.259	<0.001
Current smoking						
Never (reference) former	−0.027	0.10	−0.037	0.026	0.003	0.87
Current ≤20 cigarettes per day	−0.134	<0.001	0.039	0.018	0.081	<0.001
Current >20 cigarettes per day	−0.083	<0.001	0.025	0.076	0.068	<0.001
Alcohol consumption (g/day)						
0/rarely (reference)						
0.1–10	0.032	0.048	0.035	0.030	0.012	0.45
10–30	0.063	<0.001	0.013	0.46	−0.013	0.44
>30	0.043	0.015	0.004	0.81	−0.045	0.009
Positive anti-TPO autoantibodies (yes/no)	0.134	<0.001	−0.043	0.002	−0.024	0.059

Alcohol consumption is categorized in 0/rarely (reference category); 0.10; 10–30 and >30 g per day. β: standardized regression coefficients. All models were adjusted for BMI, diabetes, history of CVD, antihypertensive drugs, glucose-lowering drugs, lipid-lowering drugs, eGFR, UAE, total cholesterol, HDL cholesterol, triglycerides. UAE, urinary albumin excretion. TSH, triglycerides and UAE are log_e_ transformed

*anti*-*TPO* antithyroid peroxidase, *F* female, *FT*4 free thyroxine, *FT*3 free triiodothyronine, *M* male, *TSH* thyroid stimulating hormone

**Table 5:** Multivariable linear regression analyses demonstrating independent associations of thyroid function variables with urinary cotinine concentration in 5722 participants of the PREVEND study

	Model 1: TSH		Model 2: FT4		Model 3: FT3	
	β	*P*-value	β	*P*-value	β	*P*-value
Age	−0.086	<0.001	−0.106	<0.001	−0.224	<0.001
Sex (M vs. F)	0.053	0.001	−0.125	<0.001	−0.258	<0.001
Urinary cotinine	−0.122	<0.001	0.044	0.002	0.091	<0.001
Alcohol consumption (g/day)						
0/rarely (reference)						
0.1–10	0.036	0.026	0.033	0.040	0.009	0.56
10–30	0.064	<0.001	0.009	0.62	−0.015	0.37
>30	0.040	0.023	−0.001	0.96	−0.044	0.010
Positive anti-TPO autoantibodies (yes/no)	0.134	<0.001	−0.043	0.002	−0.028	0.031

Alcohol consumption is categorized in 0/rarely (reference category); 0.10; 10–30 and >30 g per day. Urinary cotinine data was measured in 5722 subjects. β: standardized regression coefficients. All models were adjusted for BMI, diabetes, history of CVD, antihypertensive drugs, glucose-lowering drugs, lipid-lowering drugs, eGFR, UAE, total cholesterol, HDL cholesterol, triglycerides. Cotinine, TSH, triglycerides and UAE are log_e_ transformed

*anti*-*TPO* antithyroid peroxidase, *F* female, *FT*4 free thyroxine, *FT*3 free triiodothyronine, *M* male, *TSH* thyroid stimulating hormone

## Discussion

In this large cohort of predominantly white European subjects selected for strict euthyroidism, we documented that current cigarette smoking is associated with modestly lower TSH and higher FT3 levels compared with never and former smokers. In univariate analyses, FT4 levels were higher in subjects who smoked ≤20 cigarettes per day vs. never and former smokers. Multivariable linear regression analyses confirmed these findings by demonstrating that TSH levels were lower, whereas FT3 levels were higher in current smokers even independent of a comprehensive number of clinical and laboratory variables including alcohol consumption. Likewise in these multivariable analyses, FT4 levels were higher in current smokers vs. never smokers. Notably, we, did not observe an incremental effect of more heavy smoking on these thyroid function parameters as determined by self-report, as judged from the standardized regression coefficients with the never smokers as reference category. In addition, our study demonstrates an inverse relationship of TSH and positive relationships with FT4 and FT3 with urinary cotinine both in univariate and in multivariable analysis. While many earlier studies have revealed that smoking is likely to affect thyroid function status in the same direction as presently shown [9–15, 27, 28], the current report represents to our knowledge the largest survey to demonstrate that these associations are independent of alcohol consumption with confirmation by urinary cotinine concentrations as objective measure of smoke exposure.

It has been suggested that smoking may influence sympathetic nervous activity and affect thyroid-directed autoimmune responses [4, 9, 10]. In the current study, pulse rate was elevated in both categories of current smokers, but systolic blood pressure and pulse pressure were not elevated. Thus, while sympathetic nervous activity is probably to some extent enhanced in smokers [29], it remains uncertain whether this would influence thyroid hormone levels. With respect to anti-TPO autoantibodies: when antibody titers were assessed as a continuous variable, higher titers were observed in smokers who smoked ≤20 and >20 cigarettes per day vs. never and former smokers. Further, the highest prevalence of positive anti-TPO autoantibodies was found in subjects who smoked >20 cigarettes per day. These findings would be consistent with an effect of smoking on thyroid-directed autoimmunity as for example widely appreciated in Graves disease and thyroid-associated ophthalmopathy [9, 10]. In comparison, previous cohort studies have variably reported increased [15] and decreased [30, 31] anti-TPO positivity in relation to smoking behavior. In this regard it is also remarkable that even under euthyroid conditions, subjects who were positive for anti-TPO autoantibodies had higher TSH, lower FT4 and also lower FT3 levels compared with anti-TPO autoantibody negative participants.

Partly opposite effects of alcohol consumption on thyroid function parameters compared with cigarette smoking were found, with higher TSH levels in subjects who reported regular alcohol consumption, and lower FT3 levels in heavy alcohol users. Hence, given that cigarette smoking may coincide with more heavy alcohol use [18, 19], the present results reinforce our assumption that it is relevant to take account of alcohol consumption when evaluating the association of smoking with thyroid function parameters. Likewise, smoking and alcohol use also affects HDL cholesterol levels in opposite directions [19, 32]. Although earlier smaller-sized studies have suggested some effects of alcohol use on thyroid function [16, 17], we are not aware of large-scale population studies on the association of alcohol consumption with thyroid function parameters. Notably, however, we could not take account of other life style factors such as exercise and nutrient intake, which my coincide with alcohol consumption.

In the interpretation of the current findings it should be considered that we defined euthyroidism by TSH, FT4 as well as FT3 levels each within the reference range making use of well-established electrochemiluminescent immunoassay systems. Our selection of euthyroid subjects based on an FT3 value within the reference range, differs from other cohort studies in which FT3 measurements were not available [7, 33]. In multivariable analysis, we observed an inverse association of TSH with age in agreement with other reports from the Netherlands [2, 34]. Of note, we also observed inverse associations of FT4 with age contrasting these earlier findings [34] and even more so with respect to FT3. These relationships were interpreted to be at least in part attributable to increasing anti-TPO positivity with age [1, 34]. The higher TSH, lower FT4 and FT3 levels in women could be ascribed to female predisposition for anti-TPO autoantibodies, although these associations were still present after adjustment for anti-TPO positively. Iodine intake may affect relationships of thyroid function parameters with age and sex [35]. In this regard it is relevant that the North of the Netherlands represents an geographic area of sufficient iodine intake, and was not identified as an area with insufficient or borderline iodine intake in the past [36].

Several other methodological aspects of our study should be acknowledged. We consider the comprehensive documentation of clinical and laboratory variables including urinary cotinine, which is considered to be the gold standard measure of cigarette smoke exposure, in a large cohort including participants with a wide age range as well as comparable participation of both sexes as a strength of our study. On the other hand, the number of participants with nonwhite ethnicity was low, hampering extrapolation to other ethnicities. Further, we carried out a cross-sectional study making that causal relationships could not be established. Nonetheless, that tobacco exposure is probably directly implicated in affecting thyroid function stems from one of the first reports on this issue which demonstrated a rise in TSH and a drop in T4 after smoking cessation [11]. Self-report on smoking behavior may result in inaccuracies consequent to smoking denial or difficulty in recalling the quantity of cigarettes smoked and exposure period [37], although good agreement with cotinine measurement was found in a previous report [38]. Such limitations could in part be responsible for the fact that we did not observe incremental effects of more heavy smoking as assessed by questionnaire on TSH, FT4, and FT3. In fact our data suggests linear relationships of smoke exposure with TSH (inverse) as well as with FT4 and FT3 (positive).

In conclusion, this large cross-sectional study among euthyroid predominantly white north European subjects demonstrates that cigarette smoking is associated with modest alterations in thyroid function as evidenced from lower TSH, higher FT4, and higher FT3 levels. These associations remain when taking account of clinical and laboratory variables including partly opposing effects of alcohol consumption.

## Supplementary Information

Supplementary Information

## Data Availability

The data that support the findings of this study are available from the corresponding author upon reasonable request.
