# Supplementary material for: Cigarette smoking is associated with higher thyroid hormone and lower TSH levels: the PREVEND study
Source: Endocrine. 2019 Nov 9;67(3):613–22. doi: 10.1007/s12020-019-02125-2 (PMC7054375; doi:10.1007/s12020-019-02125-2)
Supplement: Supplementary file 1 — Supplementary Information [file 12020_2019_2125_MOESM1_ESM.docx]

**Supplemental Table 1.** Thyroid function variables according to anti-thyroid peroxidase autoantibody status

|  | **Anti-TPO negative** (n=5317) | **Anti-TPO positive** (n=445) | *P*-value |
| --- | --- | --- | --- |
| **Women** (n; %) | 2442 (45.9) | 333 (74.8) | <0.001 |
| **TSH (**mU/L**)** | 1.54 (1.09-2.14) | 2.12 (1.40-3.01) | <0.001 |
| **FT4 (**pmol/L**)** | 15.8 ± 1.9 | 15.4 ±1.8 | <0.001 |
| **FT3 (**pmol/L**)** | 4.9 ± 0.5 | 4.8 ± 0.5 | <0.001 |

Data in mean ± SD or in median (interquartile range). Anti-TPO autoantibodies were not available 44 subjects. Abbreviations: anti-TPO, anti-thyroid peroxidase; FT3, free triiodothyronine; TSH, thyroid stimulating hormone. TSH is log_e_ transformed.
